# Gab2 deficiency prevents Flt3-ITD driven acute myeloid leukemia in vivo

**DOI:** 10.1038/s41375-021-01490-0

**Published:** 2021-12-13

**Authors:** Corinna Spohr, Teresa Poggio, Geoffroy Andrieux, Katharina Schönberger, Nina Cabezas-Wallscheid, Melanie Boerries, Sebastian Halbach, Anna L. Illert, Tilman Brummer

**Affiliations:** 1 Institute of Molecular Medicine and Cell Research, ZBMZ, Faculty of Medicine, University of Freiburg, 79104 Freiburg, Germany; 2 Faculty of Biology, University of Freiburg, Freiburg, Germany; 3 Spemann Graduate School of Biology and Medicine, University of Freiburg, Freiburg, Germany; 4 Department of Medicine I, Medical Center, University of Freiburg, Faculty of Medicine, University of Freiburg, 79106 Freiburg, Germany; 5 Institute of Medical Bioinformatics and Systems Medicine, Medical Center, University of Freiburg, Faculty of Medicine, University of Freiburg, 79106 Freiburg, Germany; 6 Comprehensive Cancer Center Freiburg (CCCF), Medical Center, University of Freiburg, Faculty of Medicine, University of Freiburg, 79106 Freiburg, Germany; 7 Max-Planck-Institute of Immunobiology and Epigenetics, 79108 Freiburg, Germany; 8 International Max Planck Research School for Molecular and Cellular Biology (IMPRS-MCB), Freiburg, Germany; 9 Centre for Integrative Biological Signaling Studies (CIBSS), 79104 Freiburg, Germany; 10 German Cancer Consortium (DKTK), Partner Site Freiburg and German Cancer Research Center (DKFZ), 69120 Heidelberg, Germany; 11 Center for Biological Signalling Studies BIOSS, University of Freiburg, 79104 Freiburg, Germany

**Keywords:** Acute myeloid leukaemia, Oncogenes, Cell signalling, Oncogenesis

## Abstract

Internal tandem duplications (ITD) of the FMS-like tyrosine kinase 3 (FLT3) predict poor prognosis in acute myeloid leukemia (AML) and often co-exist with inactivating *DNMT3A* mutations. In vitro studies implicated Grb2-associated binder 2 (GAB2) as FLT3-ITD effector. Utilizing a *Flt3-ITD* knock-in, *Dnmt3a* haploinsufficient mouse model, we demonstrate that *Gab2* is essential for the development of Flt3-ITD driven AML in vivo, as *Gab2* deficient mice displayed prolonged survival, presented with attenuated liver and spleen pathology and reduced blast counts. Furthermore, leukemic bone marrow from *Gab2* deficient mice exhibited reduced colony-forming unit capacity and increased FLT3 inhibitor sensitivity. Using transcriptomics, we identify the genes encoding for Axl and the Ret co-receptor Gfra2 as targets of the Flt3-ITD/Gab2/Stat5 axis. We propose a pathomechanism in which Gab2 increases signaling of these receptors by inducing their expression and by serving as downstream effector. Thereby, Gab2 promotes AML aggressiveness and drug resistance as it incorporates these receptor tyrosine kinases into the Flt3-ITD signaling network. Consequently, our data identify GAB2 as a promising biomarker and therapeutic target in human AML.

## Introduction

Approximately 25% of all AML carry an internal tandem duplication (ITD) in the juxtamembrane domain of the receptor tyrosine kinase (RTK) FLT3, leading to constitutive PI3K/AKT, ERK and STAT5 signaling [1–3]. Given the high frequency and prognostic impact of this mutation, several tyrosine kinase inhibitors (TKIs) targeting FLT3-ITD are now in clinical use, however, relapse due to drug resistance remains a significant problem.

The docking protein GAB2 amplifies signals downstream of cytokine and growth factor receptors, including FLT3-ITD [4]. GAB2 is recruited to activated receptors via the adaptor GRB2 and is subsequently phosphorylated at multiple tyrosine residues [5]. These modifications serve as binding sites for the tyrosine phosphatase SHP2 and the p85 subunit of PI3K, resulting in the activation of ERK and AKT/mTOR axes, respectively [5–7]. Moreover, GAB2 is implicated in STAT5 signaling [5, 8], although the exact molecular mechanisms remain unclear. Given its contribution to three oncogenic pathways, it is anticipated that GAB2 emerges as an important player in solid tumors and leukemia [9]. For example, Gab2 is an essential effector of Bcr-Abl in chronic myeloid leukemia (CML) [10–12]. The co-amplification of *GAB2* in 70% of AML/myelodysplastic syndrome patients with *MLL* amplification provided the first hint for a role of GAB2 in AML [13]. Further evidence arose from in vitro experiments in FLT3-ITD mutant human AML cell lines in which GAB2 knockdown decreased proliferation and viability [4], while increasing quizartinib sensitivity [14]. Moreover, Gab2 is required for transformation of primary murine bone marrow (BM) cells by retroviral FLT3-ITD constructs and is highly expressed in blasts of *FLT3*- and *NPM*1-mutant AML patients [14].

These findings suggest a crucial role of GAB2 in AML signaling pathways, especially as it amplifies signals from the FLT3-ITD oncoprotein and might thus contribute to the poor prognosis of this patient group and TKI resistance. However, all functional studies so far either used AML cell lines established decades ago or overexpressed FLT3-ITD in murine BM ectopically. Although the latter approach delivered novel insights, retroviral FLT3-ITD overexpression also induces transformed phenotypes distinct from AML [15]. Therefore, we studied the relevance of Gab2 in a complex in vivo setting using an autochthonous genetically engineered mouse model (GEMM) in which AML spontaneously arose by the alteration of endogenous gene loci. We demonstrate that Gab2 determines the course of AML in this GEMM as its deficiency significantly prolonged overall survival and reversed several disease symptoms. Mechanistically, we propose a novel model in which Gab2 promotes disease aggressiveness by upregulating the RTK Axl and GDNF family receptor α (Gfra2) via Stat5 signaling.

## Material and methods

### Mice

Mice (C57Bl/6 N background) were kept under specific pathogen-free conditions receiving standard diet and water *ad libitum*. Experiments were carried out in accordance with the German law for animal protection and approved by the government commission for animal protection and the local ethics committee (G-17/56; G-20/130). For genotyping primers see Supplementary methods.

### BM transplantation

Freshly isolated BM was transplanted into 8-week old male C57/Bl6N mice. Recipients underwent myeloablative body irradiation (2 × 5 Gy, 4 h apart) followed by intravenous injection of 5 × 10^6^ BM cells.

### PB analysis

PB was evaluated using a scil Vet ABC Plus+ hematology analyzer. Blood smears were examined with Pappenheim stain according to standard protocols. Results were assessed by an investigator blinded to the experimental groups.

### Histology

Paraffin-embedded sections were H&E stained according to standard protocols and imaged using a BZ9000 (Keyence) with ×20 and ×40 objectives. Images were processed and level corrected using BZ-II Analyzer software (Keyence).

### Flow cytometry

For immunophenotyping, freshly isolated cells from murine BM, spleens and PB were erythrocyte lysed, stained and analyzed using a BD LSR Fortessa. Propidium iodide (Invitrogen) was used for dead cell exclusion. For cell cycle staining, frozen BM was thawed and stained with surface antibodies. Next, cells were fixed, permeabilized and stained with an anti-Ki-67 antibody and DAPI (BioLegend). Analysis was performed using a BD LSR II Flow Cytometer. All antibodies are listed in Supplementary Methods.

### CFU assays

Frozen BM was thawed one day prior to the experiment. Then, 2.5 × 10^4^ cells per duplicate assay were plated in MethoCult GF M3434 (Stemcell Technologies). Colonies were counted after 10 days. Quizartinib (Selleckchem) was dissolved in DMSO.

### Cell culture

HL-60, KG-1a, MOLM-13, MV4–11 and THP-1 cells were a kind gift of Justus Duyster (Freiburg, Germany) and authenticated at Multiplexion (Heidelberg, Germany). Kasumi-1 cells were purchased from the German Collection of Microorganisms and Cell Cultures (DSMZ). For culture conditions see Supplementary Methods.

### Western Blotting

Western Blotting was performed as described previously [16]. Antibodies are listed in Supplementary Methods.

### RT-qPCR

Transcripts were amplified using SYBR™ Select Master Mix for CFX (Applied Biosystems) and quantified with the delta-delta Ct method normalizing to *Oaz1* expression. For primers see Supplementary Methods.

### RNA-seq

RNA was extracted from freshly isolated BM and sequenced on an Illumina HiSeq4000. Data curation was performed as described in Supplementary Methods. Results are available in the Gene Expression Omnibus (GEO) database under the accession number GSE182624 (token: mjutoqsqvhkjlsd).

### Data analysis and statistics

Statistical analysis was performed using GraphPad Prism 9. If not stated otherwise, results were compared using an unpaired t test (two-tailed). In case of three or more groups to be analyzed, a one-way ANOVA was performed. Survival curves were compared by Mantel-Cox (log-rank) test. Data are presented as mean ± SD and *p* values < 0.05 were considered statistically significant. Additional information, i.e., on tests used to correct for multiple comparisons, can be found in the Figure legends.

## Results

### Hematological analysis of Flt3-ITD/Dnmt3a double mutant mice demonstrates a profound effect of Gab2 dosage

**Fig. 1:**
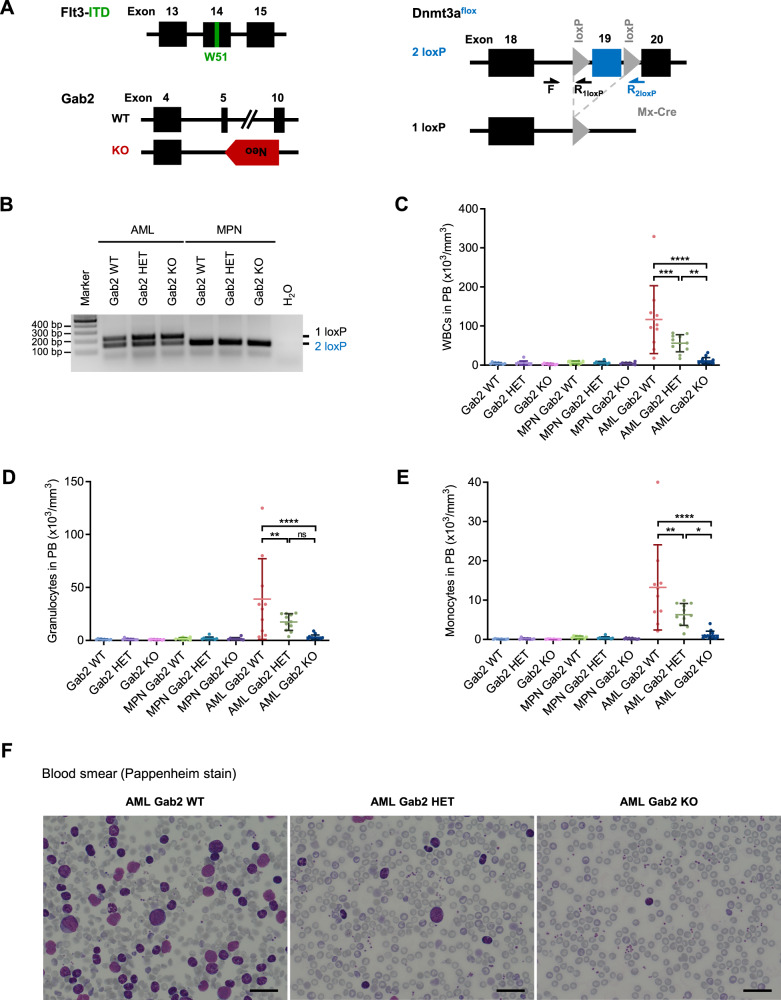
*Gab2* deficiency rescues the increased PB cell counts in a murine model of AML. **A** Schematic of the different alleles of the AML mouse model. Shown is the constitutive *Flt3-ITD* knock-in allele containing the W51 mutation in exon 14 and a *Gab2* WT as well as a KO allele with the neomycin (Neo) cassette replacing exon 5–10. In the conditional *Dnmt3a* knockout, exon 19 is flanked by two loxP sites and is deleted upon expression of Mx-Cre recombinase. Arrows indicate the binding sites for primers (F, R_1loxP_, R_2loxP_) utilized in the Dnmt3a flox PCR shown in (**B**). **B** Dnmt3a flox PCR of DNA isolated from the BM of AML and MPN mice with the indicated *Gab2* genotypes confirming the successful recombination of the *Dnmt3a* allele in AML but not in MPN mice. **C**–**E** Cell counts in the PB of mice with the indicated genotypes. Blood samples of at least 10 mice per group were evaluated. (**C**) WBC, (**D**) granulocyte, and (**E**) monocyte counts are shown. Individual values for every analyzed mouse ± SD are depicted. Statistics were calculated using one-way ANOVA with Tukey’s multiple comparison test; ns not significant; *P < 0.05; **P < 0.01; ***P < 0.001; ****P < 0.0001. Only selected significances are shown, a full list of all *P* values can be found in Supplementary Table 1. **F** Representative Pappenheim-stained blood smears of mice with the denoted genotypes. Scale bar: 100 µm. **B**–**F** All mice were aged between 21 and 30 days.

Mice harboring a *Flt3-ITD* knock-in allele only develop a mixture of myeloid and lymphoid neoplasms that do not progress into leukemia [17]. *DNA methyltransferase 3a* (*Dnmt3a*) haploinsufficiency, however, transforms Flt3-ITD mutant murine myeloproliferative neoplasms (MPNs) into AML [18–20]. Consequently, we crossed *Flt3-ITD* knock-in mice [17] with animals harboring floxed *Dnmt3a* (*Dnmt3a*^f/f^) alleles [21] and transgenic Mx-Cre mice [22] to obtain *Dnmt3a*^*f/+*^*/Flt3*^*ITD/ITD*^ as well as *Dnmt3a*^*f/+*^*/Flt3*^*ITD/ITD*^/Mx-Cre mice (Fig. 1A; crossing scheme in Supplementary Fig. 1A). The latter lose their floxed *Dnmt3a* allele upon polyinosinic-polycytidylic acid (pIpC) induced Cre expression generating *Flt3*^*ITD/ITD*^*/Dnmt3a*^*−/+*^ animals, which will from hereon be referred to as AML mice, while *Dnmt3a*^*f/+*^*/Flt3*^*ITD/ITD*^ mice that do not express Cre and therefore retain their floxed *Dnmt3a* allele will be designated as MPN mice. All animals were interbred with *Gab2* knockout mice [23] to create MPN as well as AML mice with a *Gab2*-proficient (WT), -haploinsufficient (HET) and -deficient (KO) background, respectively. This allowed for a gene dosage-dependent analysis as Gab2 HET BM expresses ~50% of Gab2 protein compared to their WT littermates [11]. Since we observed spontaneous Cre expression as indicated by recombined *Dnmt3a* alleles in all AML mice regardless of their *Gab2* genotype and without previous pIpC injections (Fig. 1B), we analyzed our mice without pIpC treatment.

First, we examined the peripheral blood (PB) in control (*Flt3*^*+/+*^*/Dnmt3a*^*+/+*^), MPN and AML mice in relation to their *Gab2* genotype. White blood cell (WBC) counts (Fig. 1C–E) were significantly increased in AML Gab2 WT (AML WT) compared to control mice regardless of their *Gab2* genotype. Strikingly, the WBC counts of *Gab2*-deficient AML (AML KO) mice were reduced and comparable to those of controls with an insignificant differential between both groups. Commensurate with their haploinsufficient genotype, AML Gab2 HET (AML HET) mice showed an intermediate phenotype with significantly higher WBCs than AML KO, but significantly lower counts than AML WT mice. These results were confirmed upon AML BM transplantation (Supplementary Fig. 1B–D). Since the analyzed mice were only 21 to 30 days old, we did not observe a blood phenotype in the MPN groups.

Importantly, AML WT mice developed anemia, a common AML symptom, while RBC counts in AML KO mice did not significantly differ from controls (Supplementary Fig. 1E). Blood smear analysis confirmed the profound differences between the *Gab2* genotypes (Fig. 1F). AML WT mice had more than 60% neoplastic cells that were either blasts or had a more differentiated myelodysplastic morphology, indicating an acute myelomonocytic leukemia. In contrast, blasts were hardly detectable in AML KO mice, while AML HET mice presented again an intermediate phenotype with few blasts in the PB. Furthermore, thrombocytes were reduced in AML WT compared to AML KO mice, which is reminiscent of thrombocytopenia in human AML.

### Gab2 deficiency protects against hepatosplenomegaly

**Fig. 2:**
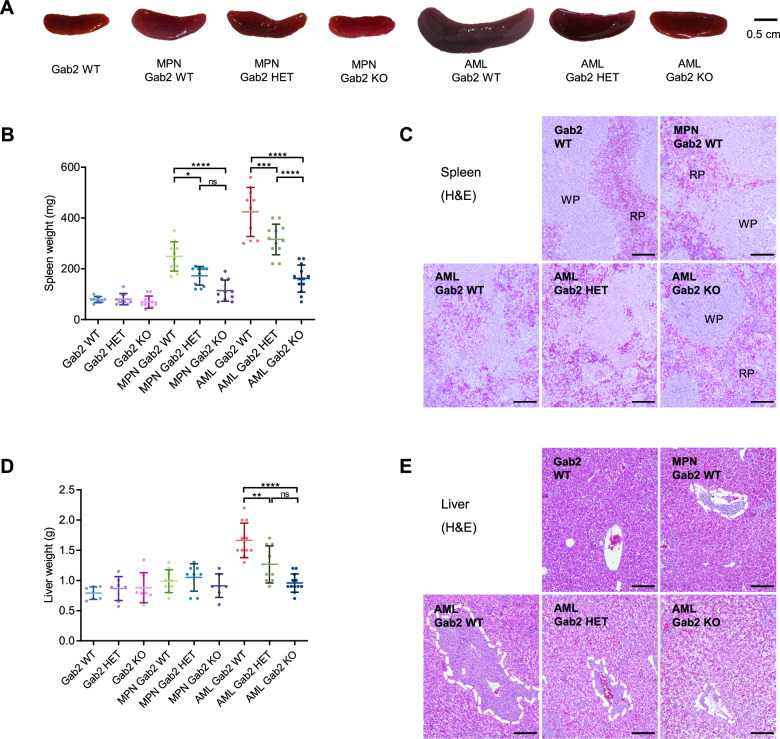
Gab2 KO protects from disease-associated changes in spleen and liver weight as well as histology in AML mice. **A** Exemplary spleen images of mice with the indicated genotypes. Effect of the *Gab2* genotype on (**B**) spleen and (**D**) liver weight of control, MPN and AML mice. Graphs show individual values for all the analyzed mice with mean ± SD. Data were analyzed using one-way ANOVA with Tukey’s multiple comparison test; ns not significant; *P < 0.05; **P < 0.01; ***P < 0.001; ****P < 0.0001. Only selected significances are indicated, a full list of all *P* values can be found in Supplementary Table 1. Representative hematoxylin and eosin (H&E) stains of (**C**) spleen and (**E**) liver sections from mice with the specified genotypes. Scale bars: 400 µm; RP = red pulp, WP = white pulp. In (**E**), perivascularily invading cells were marked with a dashed line. **A**–**E** All analyzed mice were between 21 and 30 days old.

Furthermore, we analyzed the impact of the *Gab2* genotype on BM histology (Supplementary Fig. 2A) and leukemic infiltrates as we observed markedly enlarged spleens in AML WT mice compared to controls (Fig. 2A, B, Supplementary Fig. 2B). However, spleen weight was highly significantly decreased upon Gab2 KO in AML mice and again, as observed for the blood parameters, Gab2 HET AML mice displayed an intermediate phenotype. Concerning spleen pathology, the *Gab2* genotype already had an effect in MPN mice, as the spleen weights of *Gab2*-deficient mice were significantly decreased compared to Gab2 WT mice. These results were histologically confirmed as the physiological spleen compartmentalization with clearly demarcated areas of red and white pulp observed in controls was partly disturbed in MPN and completely lost in AML WT mice (Fig. 2C, Supplementary Fig. 2A). These changes were again markedly reduced in AML KO mice. Furthermore, the effect of the *Gab2* genotype on spleen weight was recapitulated upon AML BM transplantation (Supplementary Fig. 2C).

Similarly, the increased liver weight of AML WT mice was significantly reduced in AML HET and especially in AML KO mice, in which liver weights were comparable to controls (Fig. 2D). Histological examination revealed a decreased invasion of myelomonocytic cells into perivascular and sinusoidal regions in AML HET compared to AML WT mice, which was nearly absent in AML KO mice (Fig. 2E, Supplementary Fig. 2A).

### Gab2 impacts hematopoietic stem and progenitor cell (HSPC) populations in AML mice

**Fig. 3:**
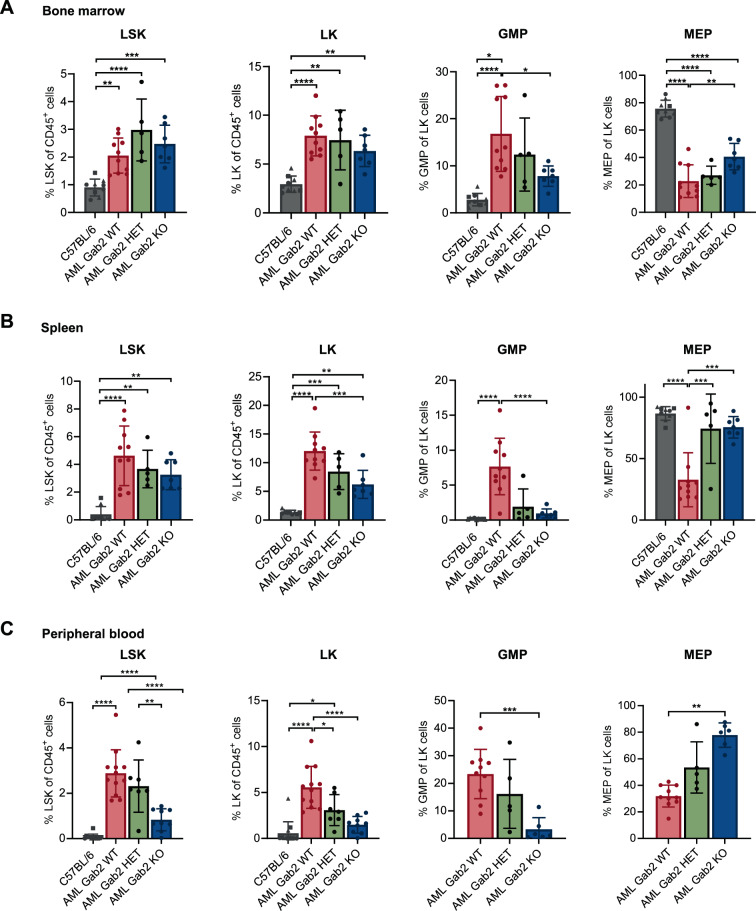
*Gab2* deficiency affects the proportion of progenitor cell populations in the PB, spleen and BM in AML mice. Flow cytometry of the (**A**) BM, (**B**) spleen and (**C**) PB of AML mice with the indicated *Gab2* genotypes. C57BL/6 mice served as healthy controls being *Gab2* proficient (circle), haploinsufficient (triangle) and deficient (square), respectively. All analyzed mice were aged between 21 and 30 days. Shown are the proportions of LSK (Lin^−^ Sca-1^+^ c-Kit^+^) and LK cells (Lin^−^ Sca-1^−^ c-Kit^+^) from CD45^+^ cells as well as the proportion of GMP (Lin^−^ c-Kit^+^ Sca-1^−^ CD34^+^ CD16/32^+^) and MEP (Lin^−^ c-Kit^+^ Sca-1^−^ CD34^−^ CD16/32^−^) cells from LK cells. Note that in (**C**) due to the very low frequency of LK cells in the PB no values for MEP and GMP cells in healthy control mice are depicted. Graphs show individual values for all the analyzed mice with mean ± SD. Statistics were calculated using a one-way ANOVA with Tukey’s multiple comparison test; *P < 0.05; **P < 0.01; ***P < 0.001; ****P < 0.0001. Representative flow cytometry blots for every analysis can be found in Supplementary Fig. 3.

Next, we characterized cell populations in the PB, BM and spleen of AML mice by immunophenotyping. In the BM, Lin^−^;Sca-1^+^;c-Kit^+^ (LSK) and Lin^−^;Sca-1^−^;c-Kit^+^ (LK) cells were increased in AML WT mice compared to healthy controls, irrespective of their *Gab2* genotype (Fig. 3A, Supplementary Fig. 3A, B). Within the LK population, an increased proportion of granulocyte-monocyte progenitor (GMP) cells was accompanied by a decrease in megakaryocyte-erythroid progenitor (MEP) cells (Fig. 3A, Supplementary Fig. 3C) comparing AML WT to control mice, thereby further confirming the myelomonocytic leukemia diagnosis. In healthy controls, the *Gab2* genotype did not affect the proportion of any of these populations (Fig. 3A), which ties in with a previous study [24]. In AML mice, however, Gab2 had profound effects on progenitor populations. Here, *Gab2* deficiency decreased the elevated proportions of GMP cells, while increasing the MEP population, thereby resembling a situation more similar to healthy controls.

Likewise, an increase in LSK cells and later stage myeloid progenitors (LK, GMP cells) was observed in the spleens and PB of AML WT mice compared to healthy controls (Fig. 3B, C). Here, the Gab2 dosage dependent rescue from this phenotype was even more evident as the size of the GMP and MEP populations in AML KO mice were similar to healthy controls. Moreover, LK cells were significantly reduced in AML KO compared to AML WT mice, while AML HET mice displayed once again an intermediate phenotype. In the PB, even the proportion of LSK cells was significantly reduced comparing AML KO mice to their AML WT littermates (Fig. 3C). Thus, the accumulation of immature cells in the periphery of AML WT mice, consistent with the observed more aggressive disease, was significantly attenuated in AML KO mice.

### *Gab2* deficiency significantly prolongs the survival of AML mice

**Fig. 4:**
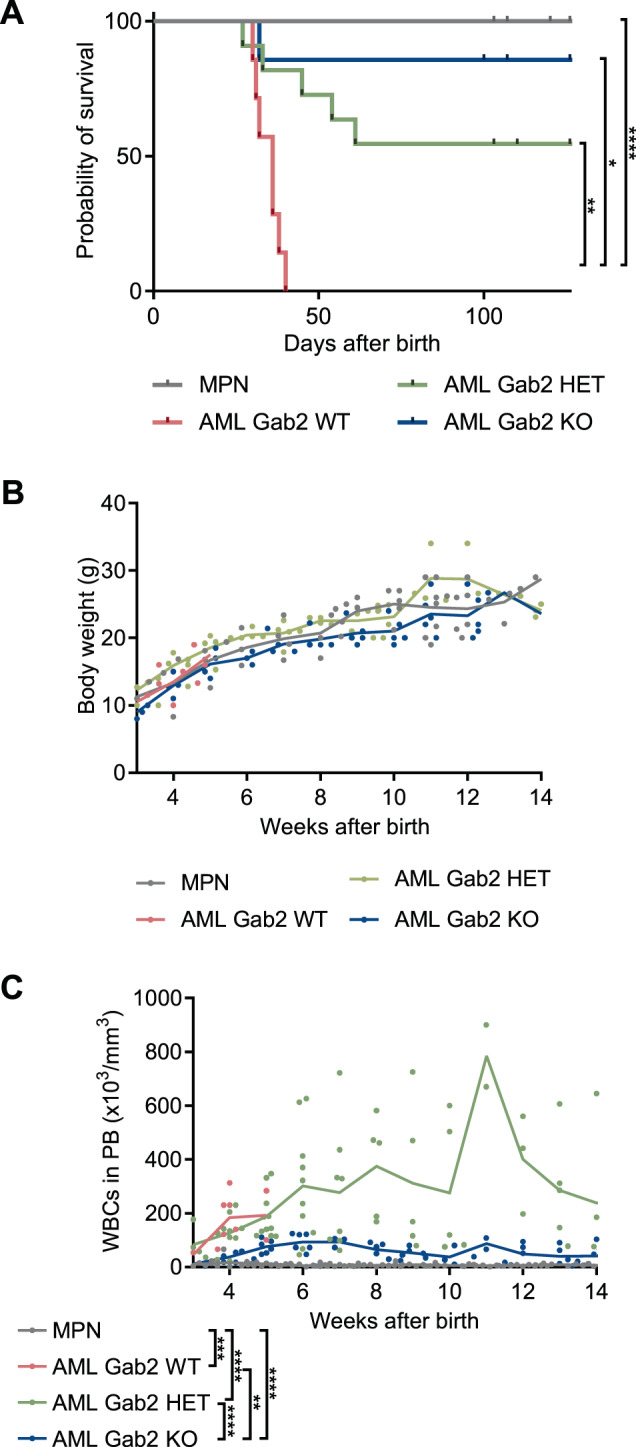
Loss of Gab2 confers a profound survival advantage. **A** Kaplan–Meier survival curves of AML WT (*n* = 7; median survival 36 days), AML HET (*n* = 11), AML KO (*n* = 7) and MPN mice (*n* = 11; including Gab2 WT, HET, and KO mice). Significant differences in survival were evaluated by Mantel–Cox (log-rank) test followed by the Bonferroni-Dunn method to adjust for multiple comparisons. Body weight (**B**) and WBC (**C**) counts in PB over time for mice with the indicated genotypes. Individual values for every measurement are shown; the connecting line indicates the mean. Data for AML WT mice are missing from week 6 onwards as all mice from this group had reached an ethical endpoint by this time. Statistics were calculated using mixed-effects analysis with Tukey’s multiple comparison test; *P < 0.05; **P < 0.01; ***P < 0.001; ****P < 0.0001.

To test whether the disease protection by *Gab2* deficiency is maintained in older mice, we monitored several mice for at least 100 days (Fig. 4A). Strikingly, while AML WT mice displayed a median survival of only 36 days, AML KO mice survived significantly longer. Here, only one mouse died spontaneously; possibly from an unrelated event. Importantly, all other AML KO mice survived the observation span without reaching an ethical endpoint. Even, AML HET mice were significantly protected, displaying again an intermediate phenotype, as 50% of the mice survived throughout the observation time. A significantly prolonged survival in mice with decreased Gab2 expression was also observed after BM transplantation (Supplementary Fig. 4A).

In addition, while the *Gab2* genotype never influenced the body weight of the respective mice (Fig. 4B), the longitudinal WBC counts reflected the aforedescribed findings: MPN mice did not develop a blood phenotype, while AML WT mice displayed highly elevated WBC counts (Fig. 4C). Likewise, AML HET mice had increased WBC counts, however, they survived considerably longer than AML WT animals. WBC counts were further reduced in AML KO mice, albeit still higher than in MPN controls. Similar findings were obtained upon BM transplantation (Supplementary Fig. 4B).

### Transcriptomic analysis of AML BM confirms profound differences between Gab2 WT and KO mice

**Fig. 5:**
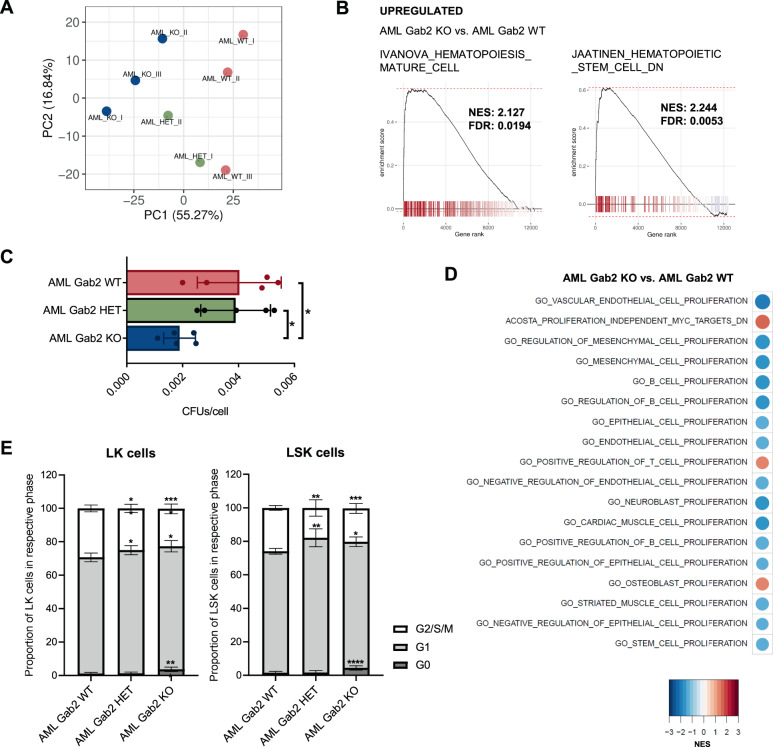
RNA-seq analysis corroborates the impact of *Gab2* deficiency on AML BM. **A** Principal component analysis was performed for RNA-seq data of whole BM isolated from AML WT, HET and KO mice, respectively. The top 10% most variable genes, based on median absolute deviation, were used. Note that the groups clearly separate according to principal component (PC) 1. **B** GSEA was carried out for AML KO versus AML WT BM. Left: Genes upregulated in mature blood cell populations are enriched in AML KO compared to AML WT BM. Right: Genes downregulated in HSCs are upregulated in AML KO versus AML WT BM. NES: Normalized enrichment score; FDR: False discovery rate. **C** CFU assays were performed in technical duplicates with BM isolated from AML WT, HET and KO mice, respectively. Bars represent mean ± SD; every dot corresponds to the BM of one particular mouse. **D** GSEA for AML KO versus AML WT BM. Shown are enrichment heat maps for gene sets with an adjusted *p* value < 0.5 containing the keyword ‘proliferation’. Color code and circle size correspond to the NES. **E** BM with the indicated genotypes was stained with Ki-67 and DAPI and subsequently subjected to cell cycle analysis via flow cytometry. G2/S/M phase was defined as Ki-67 and DAPI double-positivity, while cells in G1 phase were Ki-67 single positive and cells in G0 phase negative for both markers. Shown are the results for LK (left) and LSK (right) cells. At least 8 mice per genotype were analyzed. The experiment was repeated twice and results were pooled. Mean ± SD are shown. **C**, **E** Significance was determined by one-way ANOVA with Tukey’s multiple comparison test; *P < 0.05; **P < 0.01; ***P < 0.001; ****P < 0.0001.

Next, we performed RNA-seq of BM isolated from AML WT, HET and KO mice, respectively, to identify mechanisms explaining why *Gab2*-deficient mice are protected against AML. Consistent with the strikingly different phenotypes, the BM transcriptomes of AML WT and KO mice clearly separated in their principal components, while AML HET BM displayed an intermediate phenotype (Fig. 5A). Furthermore, we found 11 cancer-related gene sets from different tumor entities in the KEGG database downregulated in AML KO compared to AML WT mice, highlighting the strongly reduced disease aggressiveness in *Gab2*-deficient mice (Supplementary Fig. 5A).

Gene set enrichment analysis (GSEA) confirmed the immunophenotyping data, as the BM transcriptome reversed from an immature, HSC-enriched in AML WT back to a more mature, differentiated state in AML KO mice (Fig. 5B). These data were corroborated at a functional level as BM from AML KO mice formed significantly less colonies in CFU assays than AML WT mice (Fig. 5C); here, AML HET BM behaved similar to AML WT BM. This assay, together with the GSEA revealing a downregulation of proliferation-associated gene sets in AML KO BM (Fig. 5D), indicated a role for Gab2 in hematopoietic progenitor cell proliferation. This was further supported by cell cycle analysis of LK and LSK BM cells revealing significantly more LK and LSK cells in G2/S/M phase at the expense of cells in G1 phase in AML WT compared to AML HET and KO mice (Fig. 5E). In contrast, AML KO BM contained significantly increased proportions of quiescent (G0) cells. Thus, the expansion of myeloid progenitor cells in AML WT mice (Fig. 3) is, at least partially, caused by increased cell cycle progression of LK and LSK cells in the BM.

### Gab2 increases RTK signaling in AML BM and upregulates Axl as well as Gfra2 expression

**Fig. 6:**
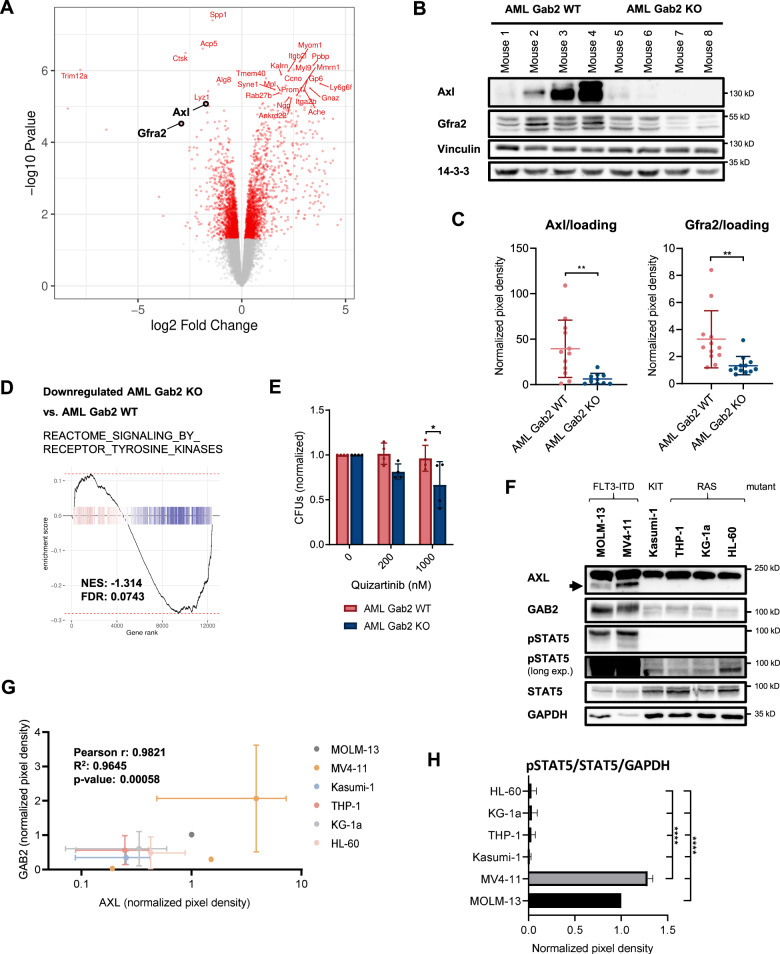
Gab2 upregulates Axl as well as Gfra2 expression and induces RTK signaling in AML BM. **A** Volcano blot of RNA-seq data comparing AML KO versus AML WT BM. Axl and Gfra2 as interesting hits are highlighted. **B** Exemplary Western Blot of murine AML WT and KO BM confirming the differential regulation of Axl and Gfra2 on protein level. Vinculin and 14–3–3 serve as loading controls. *Gab2* genotypes are confirmed in Supplementary Fig. 5B. **C** Quantification of the Western Blot analysis of Axl and Gfra2 expression in AML WT and KO BM (see also (**B**)). Protein levels were normalized to loading controls (Vinculin, 14-3-3). Each dot depicts one donor mouse. Data were analyzed using an unpaired *t* test and are presented as mean ± SD; **P < 0.01. Two Axl data points were identified as outliers using the ROUT method (Q = 1%) and excluded. Original data can be found in Supplementary Fig. 5C. **D** GSEA was performed for AML KO versus AML WT BM revealing a downregulation of genes in the REACTOME_SIGNALING_BY_RECEPTOR_TYROSINE_KINASES gene set. **E** CFU assays were performed in technical duplicates with AML Gab2 WT and KO BM, respectively. Cells were treated with the indicated quizartinib concentrations or DMSO as a control. Bars represent mean ± SD; every dot corresponds to one mouse. Data were analyzed using a one-way ANOVA with Šídák’s multiple comparisons test; *P < 0.05. **F** Exemplary Western Blot of human AML cell lines analyzing STAT5 phosphorylation as well as AXL and GAB2 expression. GAPDH serves as loading control. **G** Quantification of AXL and GAB2 expression as analyzed by Western Blot (*n* = 3; see also (**F**)). Pixel density was normalized to GAPDH loading control. Computation of Pearson correlation coefficient (r) confirmed a linear relationship between GAB2 and AXL expression in the indicated cell lines. **H** Quantification of the pSTAT5 signal in AML cell lines as analyzed by Western Blot (*n* = 3; see also (**F**)). Pixel density was normalized to STAT5 expression and GAPDH loading control. Data were analyzed using a one-way ANOVA with Tukey’s multiple comparison test and are presented as mean ± SD; ****P < 0.0001.

Among the differentially expressed genes with a large log2 fold change, we found the RTK Axl and Gfra2, a co-receptor of the RTK Ret, to be significantly decreased in AML KO compared to AML WT BM (Fig. 6A), which was confirmed at protein level (Fig. 6B, C, Supplementary Fig. 5B, C). This pinpoints to a novel role of Gab2 in which it acts not only as an amplifier of RTK signaling by being recruited to receptors, but also by inducing their expression. Accordingly, AML KO mice showed a general decrease in RTK signaling as compared to their AML WT littermates (Fig. 6D). Moreover, a phospho-RTK array analyzing the tyrosine phosphorylation of 39 different RTKs revealed increased phosphorylation of a whole variety of RTKs in AML WT compared to AML KO BM, including Axl and Ret (Supplementary Fig. 5D).

Given the growing number of reports linking AXL expression to TKI resistance in human AML [25–27] and as GAB2 confers TKI resistance in CML [11] and AML cell lines [14], we analyzed the TKI sensitivity of AML BM depending on the *Gab2* genotype. Indeed, colony formation of AML WT BM was enhanced in the presence of the FLT3 inhibitor (FLT3i) quizartinib as compared to AML KO BM (Fig. 6E). Based on this result and since AXL is increasingly gaining relevance in the context of AML [28, 29], we correlated its expression in human AML cell lines with GAB2 levels. Strikingly, AXL and GAB2 protein expression was by far the highest in the FLT3-ITD mutant MOLM-13 and MV4–11 cells (Fig. 6F). In fact, there was a highly significant linear correlation between GAB2 and AXL protein levels (Fig. 6G), suggesting a conserved role for GAB2 in inducing AXL in human AML.

### Gab2 upregulates Axl and Gfra2 via Stat5 signaling

**Fig. 7:**
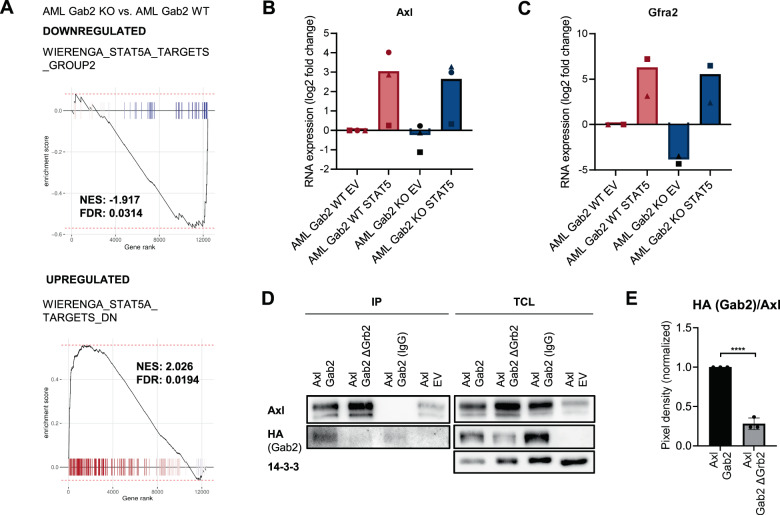
Gab2 induces Axl and Gfra2 expression *via* Stat5 pathway activation in Flt3-ITD positive AML. **A** GSEA was performed revealing a decreased Stat5 pathway activity in AML KO compared to AML WT BM. Targets that were downregulated as a result of STAT5 overexpression in human CD34+ cells were enriched (bottom), while targets that were upregulated in a linear fashion (top) were downregulated in AML KO versus AML WT BM. **B**, **C** AML WT and KO BM (4–6 mice per genotype were pooled per experiment) was retrovirally infected with pMX EV and pMX STAT5, respectively. BM was cultivated for 10 days after infection, sorted for GFP expression (pMX) and subsequently subjected to RT-qPCR. Shown is the log2 fold change of (**B**) *Axl* (*n* = 3) and (**C**) *Gfra2* (*n* = 2) RNA expression levels normalized to *Oaz1* expression. Shown are values for the individual replicates as well as the mean. Please note that in the replicate indicated by a circle, the BM was kept in culture for 1 day after sorting, while in the other replicates the BM was frozen directly after sorting. **D** HEK 293 T cells were transfected with the indicated combinations of overexpression plasmids, namely pMIG Axl, EV, Gab2 (HA) and Gab2^∆Grb2^ (HA), respectively. Subsequently, an anti-AXL immunoprecipitation (IP) was performed and the total cell lysate (TCL) as well as the IP was analyzed by Western Blotting using the indicated antibodies. 14-3-3 serves as loading control. **E** Quantification of three independent IP experiments as described in (**E**). The HA signal was normalized to the levels of precipitated AXL. Graph shows the mean value ± SD, while the dots represent individual replicates. Statistics were calculated with an unpaired *t* test; ****P < 0.01.

Recently, Dumas et al. reported AXL upregulation as a mechanism of quizartinib resistance in FLT3-ITD mutant human AML and proposed a STAT5-dependent process [25]. As GAB2 is also implicated in STAT5 activation [4, 8], we postulated a conserved FLT3-ITD/GAB2/STAT5 axis promoting AXL expression. Therefore, we examined STAT5 phosphorylation in our human AML cell line panel. Strikingly, the FLT3 mutant lines with high AXL and GAB2 expression displayed a substantially increased phosphorylation of STAT5 as compared to RAS and KIT mutant cells (Fig. 6F, H). Accordingly, GSEA analysis revealed the downregulation of STAT5 targets in AML KO compared to AML WT murine BM (Fig. 7A, top). Conversely, genes downregulated in human CD34 + cells upon STAT5 overexpression [30] were upregulated (Fig. 7A, bottom), thereby confirming decreased Stat5 signaling in *Gab2* deficient BM.

To prove that the increased *Axl* expression in AML WT mice indeed results from elevated Stat5 activity, we performed rescue experiments in which we infected BM from AML WT and KO mice with a gain-of-function (GOF) STAT5 construct (Supplementary Fig. 5E). Strikingly, *Axl* was induced upon overexpression of STAT5 in AML WT and KO BM (Fig. 7B). Unexpectedly, we also observed a strong induction of *Gfra2* (Fig. 7C), indicating that this receptor might as well be upregulated by Gab2 via Stat5 signaling. Indeed, we identified two potential SREs with the consensus sequence TTCN3GAA [31] in introns 4–5 of *Gfra2* orthologues of rats, mice and humans.

Lastly, we asked whether Gab2 also interacts with the two receptors whose expression it induces. While the interaction of Gab2 with Ret is documented [32], a Gab2/Axl interaction has not been described so far. Therefore, we confirmed their interaction in co-immunoprecipitation experiments (Fig. 7D, E). Interestingly, the interaction of AXL with the GAB2 ∆GRB2 mutant, lacking both GRB2 binding sites [16], was significantly reduced, suggesting that, like for other RTKs [5, 33], the GAB2-AXL interaction is mediated *via* GRB2.

## Discussion

In this study, we show for the first time that *Gab2* deficiency protects against AML in a spontaneous and autochthonus disease model. This GEMM closely recapitulates the human disease, as *FLT3-ITD* and *DNMT3A* mutations frequently co-exist in cytogenetically normal AML and forecast dismal prognosis [34]. Our in vivo data highlighting a critical role of Gab2 in this aggressive AML model tie in with studies describing the role of Gab2 in *Shp2/Ptpn11* mutant JMML GEMMs. Here, aberrant HSC cycling and colony formation was almost completely reversed by *Gab2* deficiency [35, 36]. However, we also noted differences: In our AML model, *Gab2* deficiency cannot attenuate the increase of LSK cells in the BM and only slightly reduced the LK population, despite its clear effect on proliferation. In the periphery, however, *Gab2* deficiency strongly reduced the proportion of LK and in the PB also of LSK cells. This suggests that the decreased proliferation in AML KO mice not directly affects the BM resident population, but primarily reduces leukemia burden by limiting the number of BM emigrating HSPCs. Supporting this, Gab2 is involved in β1-integrin signaling [37], a pathway critical for HSC homing to the BM and the retention of progenitors in the BM microenvironment [38]. Indeed, integrin-related gene sets were downregulated in AML KO versus WT BM (i.e. Integrin (ConsensusPathDB); statistical mean −1.76; *q* value 0.08 (gage)).

Interestingly, Gab2 KO mice possess the same absolute number of LK and LSK cells as Gab2 WT mice, indicating that Gab2 is dispensable for HSPC pool maintenance. However, an impaired response of *Gab2* deficient LSK cells to hematopoietic growth factors has been described [24]. This suggests that the effect of Gab2 on HSPC proliferation is not discernible at a steady-state level as here these populations are relatively quiescent. In contrast, a requirement for Gab2 might arise in the context of the increased proliferative stress in hematological disorders. This would explain the protective effect of Gab2 deficiency in our model and highlights Gab2 (and its effectors) as a disease-specific vulnerability in myeloproliferative disorders.

**Fig. 8:**
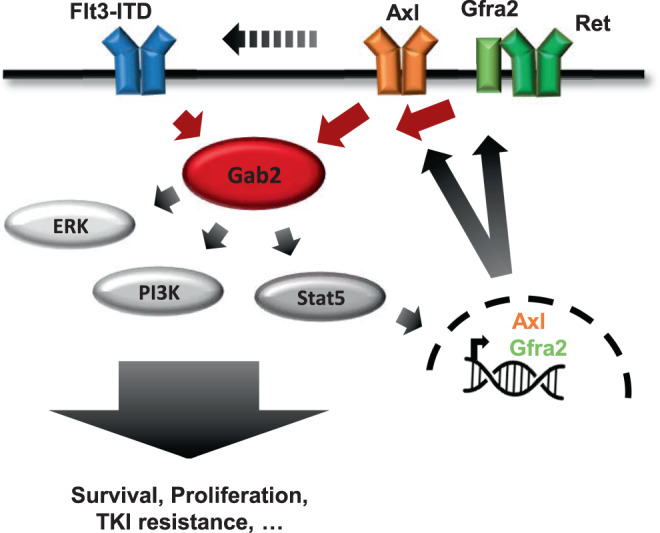
Schematic summarizing the effects of Gab2 on RTK expression and signaling in Flt3-ITD positive AML. Gab2 amplifies Flt3-ITD signaling by activating the Erk, PI3K/Akt and Stat5 pathways [4]. Stat5 pathway activation induces the expression of Axl and the Ret co-receptor Gfra2. As Gab2 also binds Axl and Ret [32], thereby being part of their downstream signaling network, the docking protein further supports the oncogenic effects of Flt3-ITD by inducing a feed-forward loop.

To unravel novel mechanisms by which Gab2 promotes AML aggressiveness, we compared the transcriptomes of AML WT and KO BM. An exciting finding, also from the angle of targeted therapy, is that Gab2 and its Stat5 effector arm act not only downstream but also upstream of RTK signaling (Fig. 8). In the following, we discuss this concept focusing on two Gab2-dependent transcriptional targets, the RTK Axl and the RTK co-receptor Gfra2.

AXL emerges as promising biomarker and therapeutic target in oncology. In AML, AXL expression correlates with dismal prognosis and therapy resistance [28, 39]. AXL phosphorylation is enhanced in FLT3i-resistant cells, while its inhibition re-sensitized cells towards FLT3i [25, 26]. Thus, the increased quizartinib resistance we observed in AML WT BM might be—at least partially—conveyed by Gab2-mediated Axl upregulation, thereby proposing a mechanism of how Gab2 contributes to TKI resistance in AML. Mechanistically, AXL might promote FLT3i resistance by acting as a bypass, since both RTKs share many pathways [40]. Here, we show that GAB2 links AXL to oncogenic pathways via a GRB2-dependent interaction. In addition, AXL might heterodimerize with other RTKs, thereby facilitating receptor activation and offsetting the effects of TKIs [41]. Indeed, Park et al. [42] reported that AXL and FLT3 physically interact in FLT3-ITD positive AML cells. Furthermore, this study suggested AXL to be crucial for FLT3 activation as its inhibition diminished FLT3-ITD phosphorylation. This does not only have implications for TKI resistance but also for AML pathobiology and FLT3 signaling in general. Since we show that Gab2, as an important Flt3-ITD effector, increases Axl expression and, as discussed, AXL in turn promotes FLT3-ITD signaling, we propose a self-sustaining feed-forward loop with a central role for Gab2 in enhancing the oncogenic signals of Flt3-ITD and contributing to AML aggressiveness (Fig. 8).

The second target of the Flt3-ITD/Gab2/Stat5 axis, Gfra2, engages in a multi-subunit complex with the RTK Ret and confers ligand binding [43]. RET is differentially expressed in human AML, with highest levels in leukemia with monocytic differentiation [44], which nicely fits to the acute myelomonocytic leukemia of our GEMM. Importantly, high RET expression in primary AML samples correlated with an adverse prognosis [45]. In contrast, *Gfra* transcripts were only rarely found in leukemic blasts, while they were—in the absence of RET—detected in BM-derived stromal cells [44, 46]. However, a leukemia intrinsic expression of the RET co-receptor is also conceivable as Rudat et al. [47] observed expression of GFRA2 (and the GFRA3 isoform) in human AML cell lines and identified a targetable co-dependency between FLT3-ITD and RET, as the latter suppressed autophagy through mTORC1 activation thereby stabilizing FLT3-ITD. In agreement with these observations and the documentation of a RET/GRB2/GAB2/PI3K/AKT/mTORC1 axis in neuronal cells [32], mTORC-related gene sets were downregulated in AML KO versus WT BM (i.e. HALLMARK_MTORC1_SIGNALING; statistical mean −1.89; *q* value 0.01 (gage)). Altogether, the mentioned studies and our present data suggest a second self-sustaining feed-forward loop in which Gab2—downstream of Flt3-ITD—increases Gfra2 expression, thereby promoting Ret activation and Flt3-ITD signaling (Fig. 8).

We propose Stat5 as the mediator by which Gab2 induces Axl and Gfra2. Despite several independent studies implicating Stat proteins as Gab2 effectors [4, 8, 48], the precise underlying molecular mechanism remains unclear. Nevertheless, several lines of evidence in the literature and generated in our study support a Flt3-ITD/Gab2/Stat5 axis. First, Flt3-ITD potently activates Stat5 [49–52]. Second, GAB2 promotes STAT5 activation downstream of FLT3-ITD [4]. Third, GAB2 promotes STAT5 activity in T cells via an ERK-mediated negative feedback [8]. Fourth, we report a reduction of the Stat5 target gene transcripts *Axl* and *Gfra2* in *Gab2* deficient Flt3-ITD expressing BM, a defect that is rescued upon ectopic expression of a GOF STAT5 mutant.

Lastly, we suggest that GAB2 and its effectors, including its druggable target gene products AXL and GFRA2/RET, should be further pursued as potential prognostic markers and therapeutic targets in AML, which could be achieved by various strategies: First, drugs in (pre)clinical development blocking the SHP2, PI3K and STAT5 axes, could be used to indirectly counteract GAB2-mediated amplification of FLT3-ITD signaling. Indeed, FLT3-driven human AML cell lines and mouse models exhibited sensitivity against STAT5 and SHP2 inhibitors, respectively [53, 54]. Furthermore, GAB2 might provide an excellent direct target in FLT3-ITD mutant AML. In contrast to the embryonic lethality displayed by *PI3K* and *Shp2* deficient mice [55, 56], *Gab2* KO mice are viable and fertile without overt phenotypes [23, 57]. Thus, blocking GAB2 might specifically impair leukemia cells with considerably less side effects than targeting its more ubiquitously expressed effectors PI3K and SHP2.

By inducing and interacting with RET and AXL (and potentially additional RTKs), GAB2 could alleviate FLT3-ITD addiction and thereby contribute to FLT3 inhibitor resistance. Blocking GAB2 function or expression would be superior to targeting FLT3 directly, since the docking protein serves downstream of various leukemia-relevant RTKs. In that regard, GAB2 deficiency could mimic, at least in part, the effects of the AML relevant multi-kinase inhibitors sorafenib and midostaurin, while more selective drugs such as quizartinib rather imitate FLT3-ITD deficiency [58]. Moreover, inhibiting GAB2 would also be applicable in case of FLT3 inhibitor resistance, including mechanisms like FLT3 point mutations, amplifications or the upregulation of additional RTKs. However, as GAB2 lacks intrinsic enzymatic activity, new approaches will be required such as blocking the GAB2/GRB2 interaction, either directly [59] or indirectly by stabilizing the inhibitory GAB2/14-3-3 complex [11, 60], a strategy for which general feasibility has been demonstrated [61].

## Supplementary information

Supplementary Methods and Figures

Supplementary Table
