# Supplementary material for: Gab2 deficiency prevents Flt3-ITD driven acute myeloid leukemia in vivo
Source: Leukemia. 2021 Dec 13;36(4):970–82. doi: 10.1038/s41375-021-01490-0 (PMC8979819; doi:10.1038/s41375-021-01490-0)
Supplement: Supplementary file 1 — Supplementary Methods and Figures [file 41375_2021_1490_MOESM1_ESM.pdf]

Supplementary Information for

Gab2 deficiency prevents Flt3-ITD driven acute myeloid leukemia *in vivo*

Corinna Spohr, Teresa Poggio, Geoffroy Andrieux, Katharina Schönberger, Nina Cabezas-Wallscheid, Melanie Börries, Sebastian Halbach, Anna L. Illert, Tilman Brummer*

*Corresponding author: tilman.brummer@mol-med.uni-freiburg.de

This PDF file includes:

1. Supplementary Methods

2. Supplementary Figure 1

3. Supplementary Figure 2

4. Supplementary Figure 3

5. Supplementary Figure 4

6. Supplementary Figure 5

7. Supplementary References

1. Supplementary Methods

Mouse genotyping

Mice were genotyped by PCR using the following primer pairs:

**Allele**	**Primer**	**Sequence**
Gab2	Gab2 WT 1	AACTACGTGCCCATGAACCCAGGTTC
	Gab2 WT 2	AGATGGGGCAAAAAGTTGGCTTACC
	Gab2 MUT 1	CGCCTACCGGTGGATGTGGAATGTG
	Gab2 MUT 2	CCCTGGGTTATCATTCTCCGCTGGG
Flt3-ITD	FLT3 9854	TCTGGTTCCATCCATCTTCC
	FLT3 WT 9855	AGGAAGTCGATGTTGGCACT
	FLT3 Mut oIMR6773	TGGCTACCCGTGATATTGCT
Mx-Cre	CreA	GCATAACCAGTGAAACAGCATTGCTG
	CreB	GGACATGTTCAGGGATCGCCAGGCG
	Taconic 1260-1	GAGACTCTGGCTACTCATCC
	Taconic 1260-2	CCTTCAGCAAGAGCTGGGGAC
Dnmt3a	Dnmt3a-F	TGGGGATTTGAGAGGTGAAG
	Dnmt3a-R	GTGGAGCACTGAACAGCAAG
Dnmt3a flox	Dnmt3a LoxP-F	TGGGGATTTGAGAGGTGAAG
	Dnmt3a 2LoxP-R	AAGCCTCAGGCCCTCTAGGCAAGAT
	Dnmt3a 1LoxP-R	TGAGTGGTGAGGCCCAGCTTATCGA

Flow cytometry antibodies

All flow cytometry antibodies were purchased from eBioscience and BioLegend.

**Antigen**	**Clone**
CD4	GK1.5
CD8a	53-6.7
CD11b	M1/70
CD16/32	93
CD34	RAM34
CD45	30-F11
CD45R	RA3-6B2
CD90.2	53-2.1
CD117	2B8
CD127	SB/199
Gr-1	RB6-8C5
Ki-67	16A3
Sca-1	D7
Ter-119	TER-119

Cell culture media

**Cells**	**Medium**
HL-60<br>MOLM-13<br>THP-1	RPMI<br>10% FCS<br>2 mM L-glutamine<br>10 mM HEPES<br>100 U/ml penicillin

	100 µg/ml streptomycin.
KG-1a<br><br>Kasumi-1	RPMI<br><br>20% FCS<br><br>2 mM L-glutamine<br><br>10 mM HEPES<br><br>100 U/ml penicillin<br><br>100 µg/ml streptomycin.
MV4-11	IMDM<br><br>10% FCS<br><br>2 mM L-glutamine<br><br>10 mM HEPES<br><br>100 U/ml penicillin<br><br>100 µg/ml streptomycin.
Primary BM	IMDM<br><br>20% FCS<br><br>10 ng/ml of IL-3<br><br>10 ng/ml IL-6<br><br>10 ng/ml SCF<br><br>2 mM L-glutamine<br><br>10 mM HEPES<br><br>100 U/ml penicillin<br><br>100 µg/ml streptomycin.

Western Blotting antibodies

Antigen	Clone	Dilution	Company
14-3-3	sc-1657	1:1000	Santa Cruz
Axl	sc-1097<br>sc-166269	1:1000	Santa Cruz
Gab2	#3239	1:1000	Cell Signaling
GAPDH	ab9489	1:2000	abcam
Gfra2	ab8027	1:1000	abcam
HA	3F10	1:2000	Roche
phospho-STAT5 Tyr694	#9351	1:1000	Cell Signaling
STAT5	#9363	1:1000	Cell Signaling
Vinculin	#4650	1:1000	Cell Signaling

RT-qPCR primers

Primer	Sequence
Axl forward<br>Axl reverse	5’-TGAGCCAACCGTGGAAAGAG-3’<br>5’-AGGCCACCTTATGCCGATCTA-3’
Gfra2 forward<br>Gfra2 reverse	5’-AGTTGCTCCTATGAGGACAAGGAGAAGC-3’<br>5’-ATAGATGTGCAGGTGGTGATGACACTGG-3’
Stat5b forward<br>Stat5b reverse	5’-GCACCTTCAGATCAACCAAAC-3’<br>5’-CAGCTGGGCAAACTGAG-3’
Oaz1 forward<br>Oaz1 reverse	5’-TTTCAGCTAGCATCCTGTACTCC-3’<br>5’-GACCCTGGTCTTGTCGTTAGA-3’

Expression vectors

pMIG-Axl was generated by subcloning the Axl cDNA from pDONR 233-Axl (kind gift from William Hahn, Dana Farber Cancer Institute; Addgene plasmid #23945) into pMIG-GW (kind gift from Liz Caldon, Garvan Institute) using Gateway technology. pMIG-Gab2-HA and Gab2ΔGrb2-HA were described previously (15). pMX-EV and pMX-STAT5B, encoding a GOF STAT5 protein (H299R, S711F), were a kind gift from Robert Zeiser (University of Freiburg, Germany).

RNA-seq

Paired-end reads were first trimmed, with Trimmomatic (1), to remove adapter content and bad quality reads. Alignment and read-per-gene quantification were done with STAR (2). Differential analysis was performed with limma R package (3). Adjusted pvalue (Benjamini-Hochberg) below 0.05 was considered as significant. The fgsea R package was used to test the enrichment of gene sets coming from MSigDB (4) and ConsensusPathDB (5). Adjusted pvalue below 0.05 was considered as significant.

2. Supplementary Figure 1

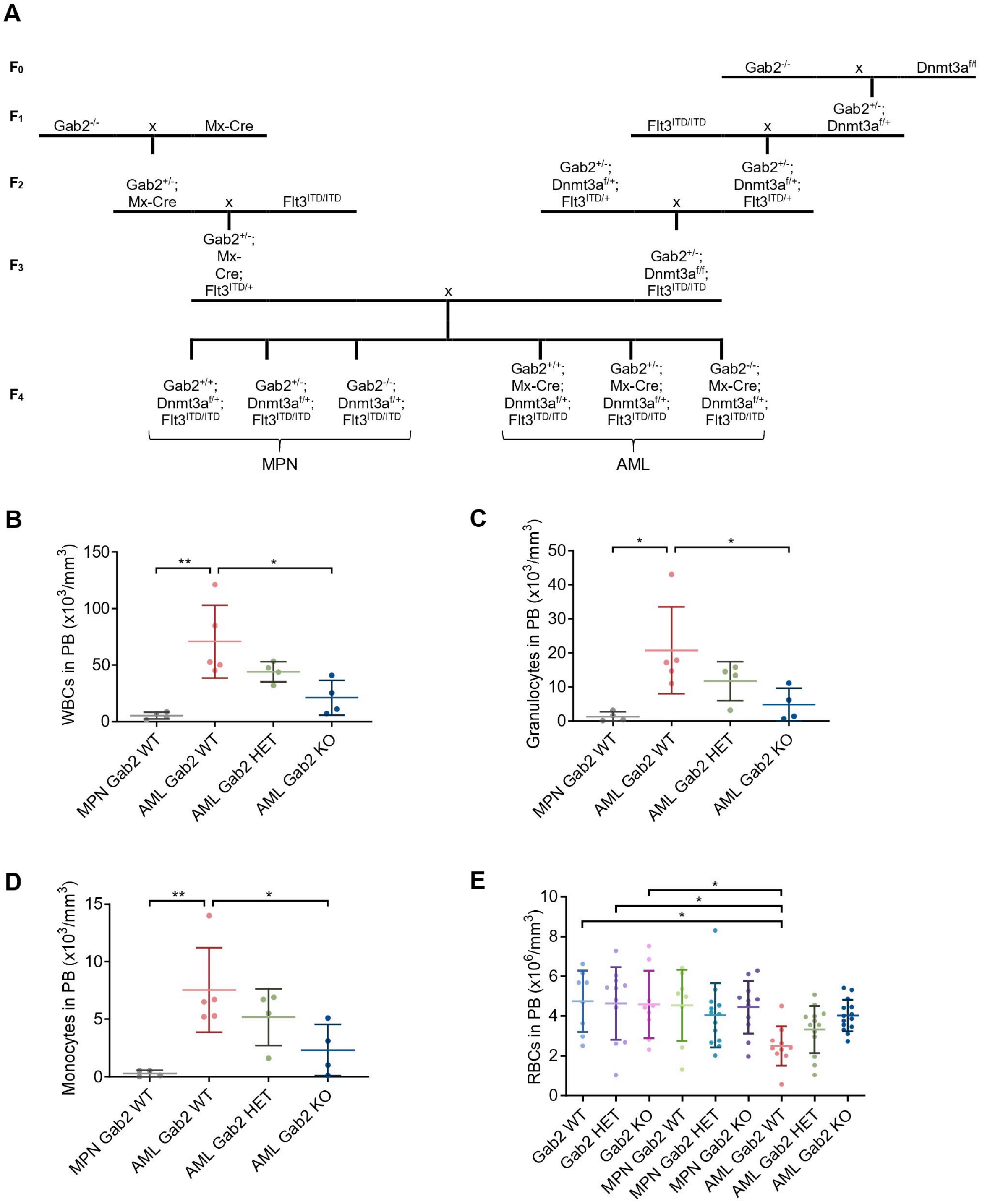

Supplementary Fig. 1 Gab2 deficiency rescues the increased PB cell counts in a primary AML mouse model as well as after BM transplantation. A Crossing scheme showing the breeding of Dnmt3a$^{f/+}$; Flt3$^{ITD/ITD}$ (MPN) and Mx1-Cre; Dnmt3a$^{f/+}$; Flt3$^{ITD/ITD}$ (AML) mice that are Gab2 proficient (Gab2$^{+/+}$), haploinsufficient (Gab2$^{+/-}$) and deficient (Gab2$^{-/-}$), respectively. **B-D** Whole BM with the indicated genotypes was isolated from 23 day old mice of the primary model and transplanted into 4 to 5 irradiated C57BL/6 recipients. Cell counts in the PB were analyzed at day 26 after transplantation. Every dot corresponds to one recipient mouse. (B) WBC, (C) granulocyte and (D)

monocyte counts are shown. **E** Red blood cell (RBC) counts in the PB of mice of the primary model. Mice aged between 21 and 26 days were evaluated. **B-E** Individual values including mean and SD are depicted. Statistics were calculated using one-way ANOVA with Tukey's multiple comparison test; * $P < 0.05$; ** $P < 0.01$.

3. Supplementary Figure 2

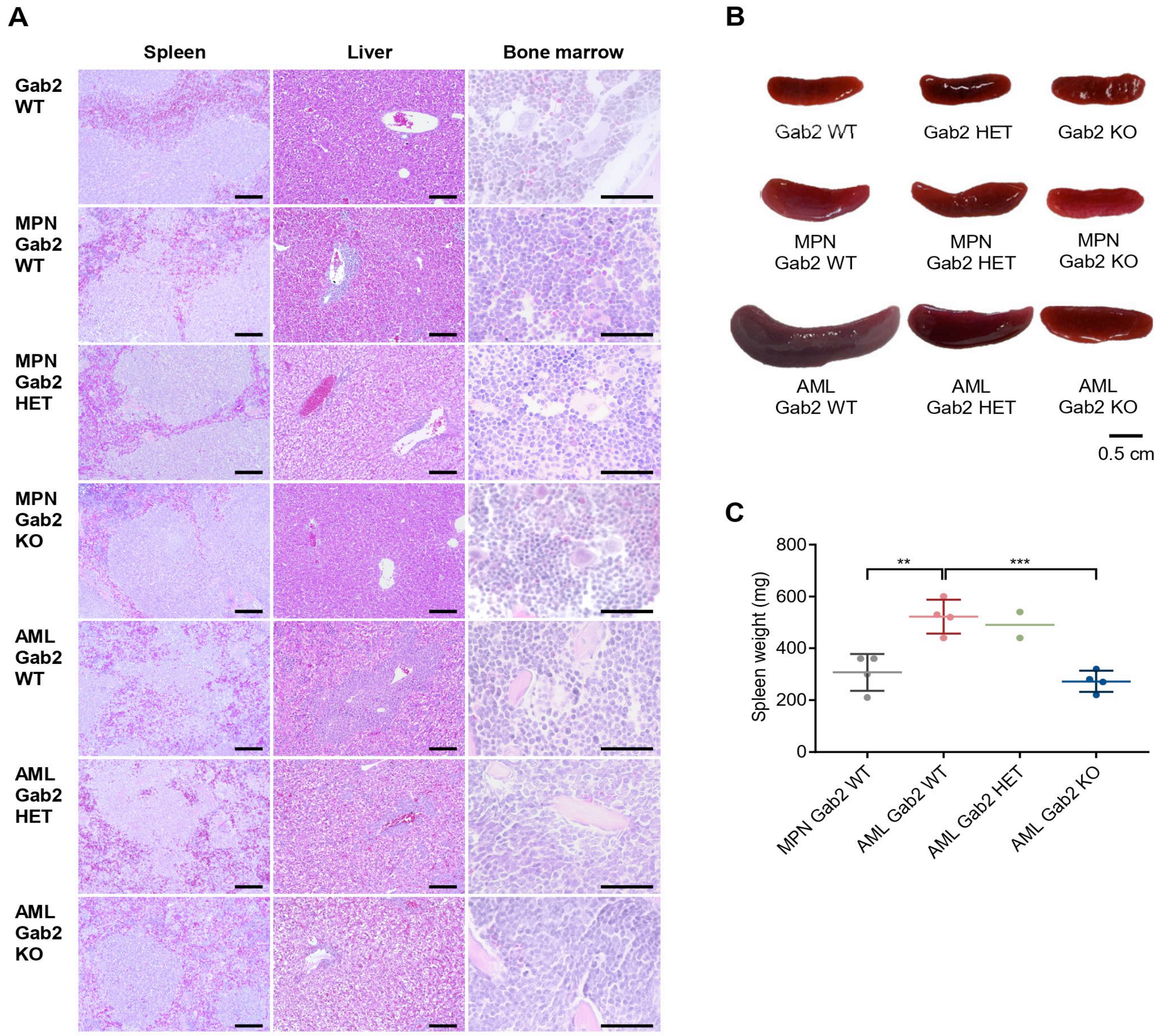

Supplementary Fig. 2 Gab2 KO protects from disease-associated changes in the spleens of a primary murine AML model as well as after transplantation. A Representative hematoxylin and eosin (H&E) stains of spleen (left; scale bar: 400 µm), liver (middle; scale bar 400 µm) and BM (right; scale bar 50 µm) sections from mice with the denoted genotypes. For liver and spleen, the same pictures as in Fig. 2C are shown, but the panel was extended with images from MPN Gab2 HET and KO mice. **B** Exemplary spleen images of control, MPN and AML mice with the indicated *Gab2* genotypes. This figure resembles Fig. 2A, but in addition shows spleen images of conditions that were excluded from the main figures because of space limitations. **A, B** All analyzed mice were between 21 and 30 days old. **C** Effect of the *Gab2* genotype on spleen weights of mice transplanted with BM isolated from 23 day old mice of the primary AML model. BM of one donor mouse per genotype was transplanted into 4 to 5 recipients. The graph shows individual values for all the analyzed mice with mean ± SD. Data were evaluated using one-way ANOVA with Tukey's multiple comparison test; ** $P < 0.01$; *** $P < 0.001$.

4. Supplementary Figure 3

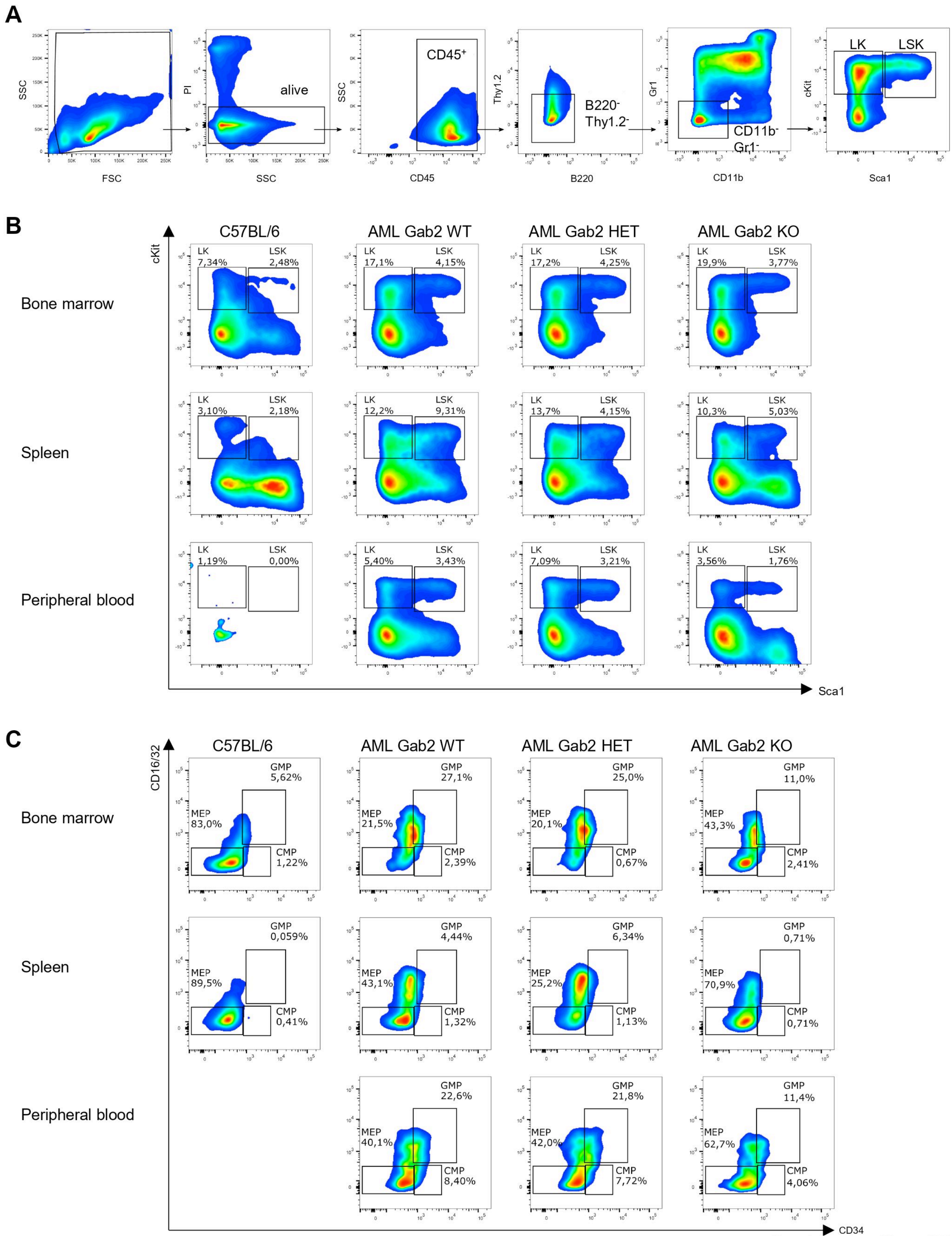

Supplementary Fig. 3 Exemplary flow cytometry blots and gating schemes for the immunophenotyping of AML mice. A Representative dot plots showing the gating strategy. **B** Exemplary dot plots of LK and LSK cells gated on CD45^{+} cells for BM, spleen and PB of mice with the

indicated genotypes. **C** Representative dot plots of GMP, MEP and common myeloid progenitor (CMP) cells gated on LK cells for BM, spleen and PB of mice with the indicated genotypes.

5. Supplementary Figure 4

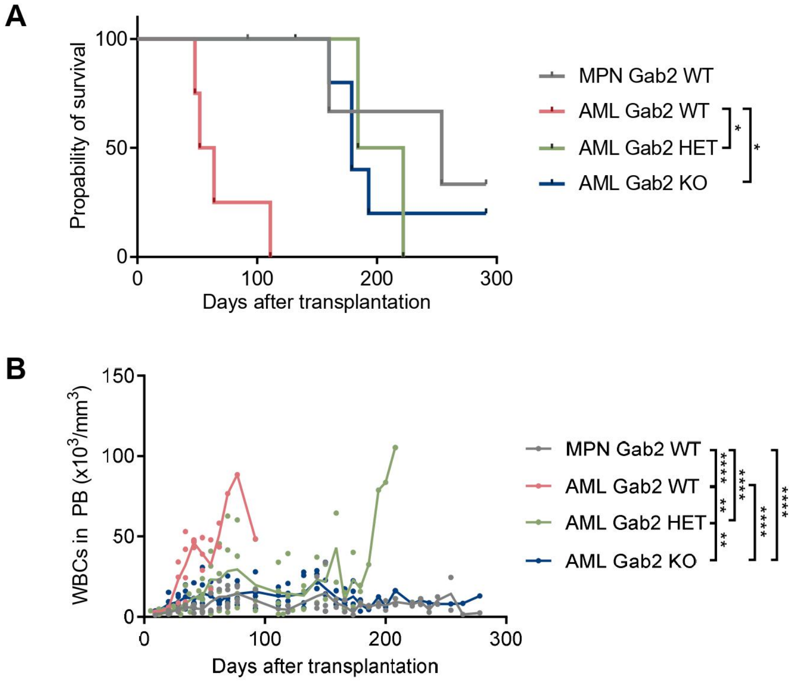

Supplementary Fig. 4 The survival advantage of Gab2 deficient and haploinsufficient AML mice over Gab2 WT AML mice is confirmed upon BM transplantation. A Kaplan-Meier survival curves of irradiated wild type mice after secondary transplantation with BM isolated from AML WT (n = 4; median survival 58 days), AML HET (n = 4; median survival 203 days), AML KO (n = 5, median survival 179 days) and MPN mice (n = 4; median survival 254 days). Significant differences in survival were evaluated by Mantel–Cox (log-rank) test followed by the Bonferroni-Dunn method to adjust for multiple comparisons; * Padj < 0.05. **B** WBC counts in the PB over time for mice after secondary BM transplantation from donors with the indicated genotypes. Every individual measurement is shown while the connecting line indicates the mean. Statistics were calculated using mixed-effects analysis with Tukey's multiple comparison test; ** P < 0.01; **** P < 0.0001.

6. Supplementary Figure 5

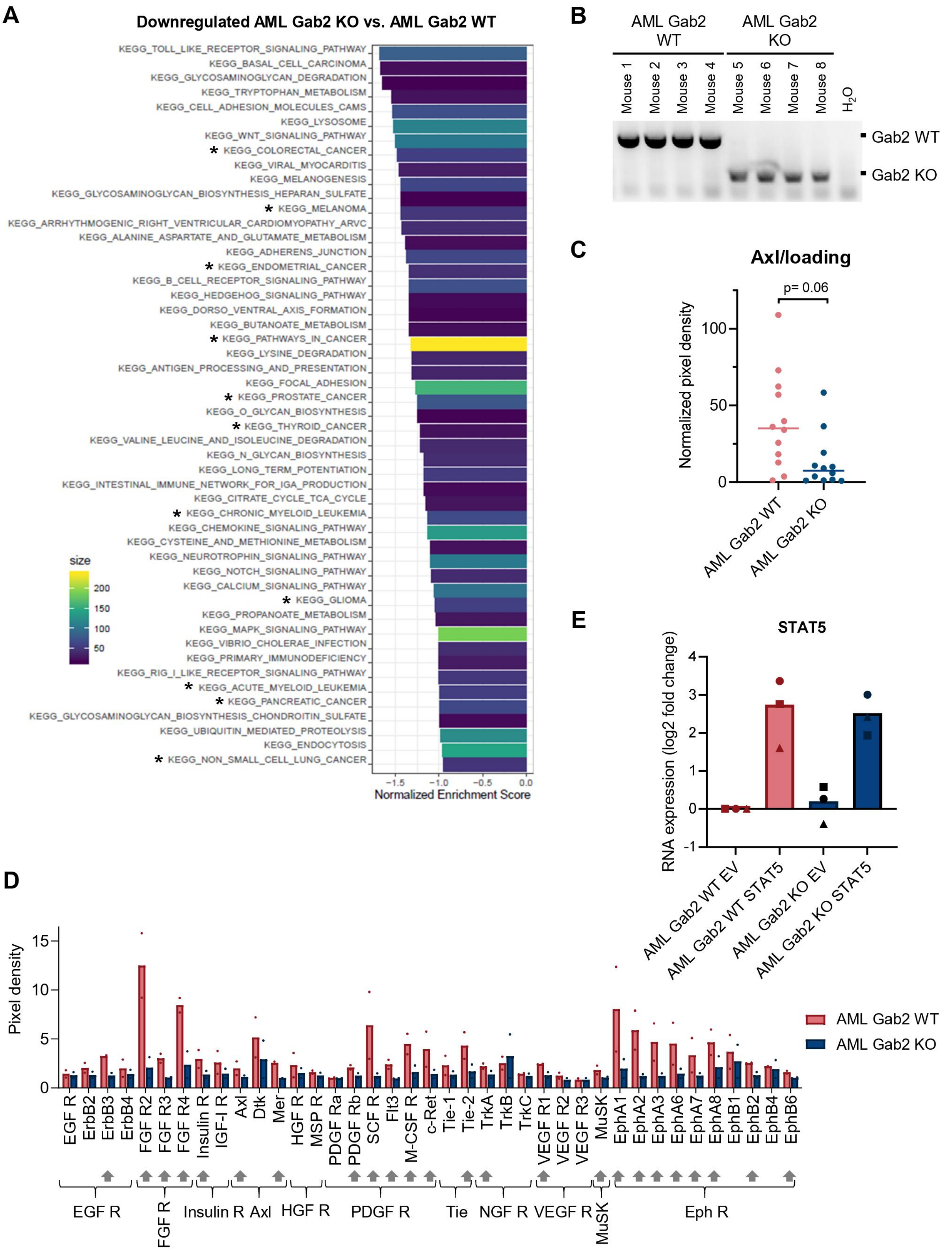

Supplementary Fig. 5 Gab2 is important for RTK signaling and induces Axl and Gfra2 expression *via* Stat5 pathway activation in Flt3-ITD positive AML. A GSEA was performed for the calculated log2 fold change between AML KO and AML WT BM. Bar plot shows the top 50 downregulated pathways

of the Kyoto Encyclopedia of Genes and Genomes (KEGG) database sorted by NES. Color code represents the number of genes included in the respective gene sets. Gene sets associated with different tumor entities are highlighted with an asterisk. **B** PCR analysis confirming the *Gab2* genotype of the mice whose BM was used for the Western Blot shown in Fig. 6B as Gab2 was not detectable on protein level *via* Western Blot in freshly isolated BM. **C** Quantification of the Western Blot analysis of Axl expression in AML WT and KO BM without outlier exclusion (see also Fig. 6B, C). Protein levels were normalized to the respective loading controls (Vinculin, 14-3-3). Each dot depicts one donor mouse. **D** Quantification of a phospho-RTK array (ARY014; R&D systems) performed with BM isolated from AML WT and AML KO mice (n = 2/genotype; individual values and mean are shown). Note the increased phosphorylation of a variety of RTKs in the WT condition. Arrows indicate receptors that were consistently higher phosphorylated in both AML WT BM lysates as compared to both AML KO lysates. Brackets summarize the indicated RTK families. **E** AML WT and AML KO BM (4 - 6 mice per genotype were pooled per experiment) was retrovirally infected with pMX EV and pMX STAT5, respectively. The latter construct encodes for a GOF mutant of STAT5. BM was cultivated for 10 days after infection and sorted for GFP expression (pMX). Shown are the results of an RT-qPCR for *STAT5* confirming its overexpression compared to EV controls. *STAT5* expression levels were normalized to the expression of the housekeeping gene *Oaz1*. Please note that in the replicate indicated by a circle the BM was kept in culture for 1 day after sorting, while in the other two replicates the BM was frozen directly after sorting.

7. Supplementary References

1. Bolger AM, Lohse M, Usadel B. Trimmomatic: a flexible trimmer for Illumina sequence data. Bioinformatics. 2014;30(15):2114-20.
2. Dobin A, Davis CA, Schlesinger F, Drenkow J, Zaleski C, Jha S, et al. STAR: ultrafast universal RNA-seq aligner. Bioinformatics. 2013;29(1):15-21.
3. Ritchie ME, Phipson B, Wu D, Hu Y, Law CW, Shi W, et al. limma powers differential expression analyses for RNA-sequencing and microarray studies. Nucleic Acids Res. 2015;43(7):e47.
4. Subramanian A, Tamayo P, Mootha VK, Mukherjee S, Ebert BL, Gillette MA, et al. Gene set enrichment analysis: a knowledge-based approach for interpreting genome-wide expression profiles. Proc Natl Acad Sci U S A. 2005;102(43):15545-50.
5. Kamburov A, Stelzl U, Lehrach H, Herwig R. The ConsensusPathDB interaction database: 2013 update. Nucleic Acids Res. 2013;41(Database issue):D793-800.
